# Effects of Capacitive and Resistive Energy Transfer Therapy (TECAR) and Low-Level Laser Therapy (LLLT) on Blood Pressure During the Musculoskeletal Treatment: A Preliminary Study

**DOI:** 10.7759/cureus.98502

**Published:** 2025-12-05

**Authors:** Ana-Maria Fatu, Roxana Oana Ciobotaru, Aurelia Romila, Claudiu Elisei Tanase

**Affiliations:** 1 Biomedical Sciences, "Dunarea de Jos" University of Galați, Galați, ROU; 2 Cardiology, "Dunarea de Jos" University of Galați, Galați, ROU; 3 Physical Medicine and Rehabilitation, "Dunarea de Jos" University of Galați, Galați, ROU

**Keywords:** hypertension, laser, rehabilitation, tecar, treatment

## Abstract

Introduction: The systemic effects of capacitive and resistive energy transfer therapy (TECAR) and low-level laser therapy (LLLT) on hemodynamic parameters remain insufficiently documented. Although these procedures are known for their local impact on microcirculation, there is no solid clinical evidence regarding their influence on blood pressure. This study aims to describe the evolution of systolic and diastolic blood pressure during a standardized therapeutic protocol and to evaluate the safety of these interventions in patients with musculoskeletal disorders, including those with treated hypertension.

Materials and methods: A prospective observational study with repeated measurements was conducted across two clinical centers in Galați, Romania, between October 2023 and June 2025. A total of 268 patients with clinically and imaging-confirmed musculoskeletal disorders were included. Systolic and diastolic blood pressure were measured before and after TECAR and LLLT at three evaluation points (day 1, day 5, and day 10). Statistical analysis employed repeated-measures analysis of variance (ANOVA) and linear mixed models to compare responses between hypertensive and normotensive patients.

Results: Statistical analyses revealed a discrete yet statistically significant reduction in systolic blood pressure at specific points within the therapeutic protocol. The Kolmogorov-Smirnov test indicated deviations from normality for post-procedure systolic values on days 1 and 5 following TECAR therapy (p = 0.010; p < 0.001), and on day 10 after LLLT (p = 0.044), suggesting increased variability in systolic responses at these stages. Comparisons between hypertensive and normotensive patients showed similar overall response patterns. Although initial sessions exhibited small group-dependent fluctuations, these differences progressively diminished, and systolic values converged by day 10, indicating a comparable hemodynamic profile and preserved safety across both categories. Diastolic blood pressure remained stable throughout the intervention, with no significant changes detected between pre- and post-session measurements. Repeated-measures ANOVA confirmed significant variations only in systolic values, while diastolic measurements showed no statistically relevant fluctuations.

Conclusion: This study showed that TECAR and LLLT are cardiovascularly well tolerated, producing only minimal and transient variations in blood pressure, with modest statistically significant reductions in systolic values and no significant changes in diastolic pressure. Responses were similar in hypertensive and normotensive patients, supporting an adequate safety profile. Controlled clinical studies are needed to further clarify any potential systemic hemodynamic effects of these therapies.

## Introduction

Musculoskeletal disorders represent a major public health concern, affecting a substantial number of adults worldwide [1]. Arterial hypertension (HTN) is frequently associated with these conditions and contributes to increased cardiovascular risk. In this context, rehabilitation strategies must address both the improvement of musculoskeletal symptoms and the monitoring of potential hemodynamic variations that may occur during treatment [1-3].

Capacitive and resistive energy transfer therapy (TECAR) [4,5] and low-level laser therapy (LLLT) [6] exhibit well-documented localized physiological effects, particularly through the modulation of microcirculation and tissue metabolic activity. Both modalities have been shown to induce regional vasodilation, enhance blood flow and tissue oxygenation, and modulate endothelial function, while concurrently attenuating local inflammation via reductions in pro-inflammatory cytokines [5-7]. LLLT mediates these responses primarily through nitric oxide release and the promotion of cellular regeneration, whereas TECAR acts predominantly through thermogenic and bioelectrical mechanisms that augment local perfusion [4,7].

Although such mechanisms could theoretically contribute to reductions in peripheral vascular resistance, current evidence indicates that these responses remain confined to the treated area, with no consistent demonstration of systemic effects on arterial blood pressure. At present, high-quality clinical studies assessing systemic hemodynamic changes following TECAR or LLLT remain limited, and the available literature is largely derived from experimental investigations and animal models. Consequently, any potential systemic hemodynamic influence of these therapies remains insufficiently substantiated by clinical evidence. In the absence of robust clinical evidence, evaluating blood pressure dynamics in the context of these procedures becomes essential.

Therefore, the present study aims to analyze blood pressure variations before and after treatment in order to determine the safety profile of these modalities, identify potential systemic effects, and support the adaptation of physiotherapeutic management for patients with cardiovascular comorbidities. Based on the local physiological mechanisms described in the literature, we hypothesized that TECAR therapy and/or LLLT may induce a modest yet detectable reduction in blood pressure after 10 treatment sessions, while maintaining an adequate safety profile in hypertensive patients receiving pharmacological therapy.

Integrating these modalities into rehabilitation programs may contribute to the development of more comprehensive therapeutic strategies, with potential benefits not only for musculoskeletal recovery but also for patients’ cardiovascular profile [8].

## Materials and methods

This was a prospective, observational, longitudinal, multicenter clinical study with repeated measures, conducted at the “St. John” Emergency Clinical Hospital for Children, Galați, and the Reumadiagnostic Clinic, Galați, Romania, between October 2023 and June 2025. A total of 268 patients with clinically and imaging-confirmed musculoskeletal disorders were included.

Inclusion criteria

Adults over 18 years of age presenting with musculoskeletal symptoms that necessitated the use of combined TECAR and LLLT therapy, and who provided written informed consent for the use of their personal data and participation in the research protocol, were included.

Exclusion criteria

Patients were excluded if they exhibited any absolute contraindications to physiotherapy, TECAR, or LLLT, including pregnancy, neoplasms, epilepsy, electronic implants, hypertensive crises, local infections, acute inflammations, decompensated cardiopulmonary conditions, acute venous thrombosis, coagulation disorders, hemophilia, use of photosensitizing medications, or systemic lupus erythematosus.

Treatment protocol

At the Reumadiagnostic Center, LLLT was delivered using the BTL 4000 Premium device (BTL Industries, Prague, Czech Republic) in combination with targeted radiofrequency (TR) therapy, BTL 6000 (BTL Industries), applied in dynamic mode. In the Rehabilitation Department of the “St. John” Emergency Clinical Hospital for Children, Galați, the same BTL unit with LLLT was employed together with CareTherapy (Fisiomed, Mussolente, Italy), applied in static mode. The BTL LASER probe emitted infrared radiation with a wavelength of 830 nm and a power of 400 W, and the parameters were chosen depending on the pathology, according to the recommendations of WALT and the manufacturing company. Thus, at the spinal level, eight application points were used, each delivering 5 J/cm^2^; for large joints, 5 points were applied with 5 J/cm^2^ for the knee and ankle, 4 J/cm^2^ for the shoulder, and 6 J/cm^2^ for the hip, while for the hand, only 2 points were used, each delivering 3 J/cm^2^. The application time was one minute per point, and the frequency was adjusted according to the type of pain: 10 Hz for acute pain and continuous frequency for chronic pain. The radiofrequency therapy was standardized and applied identically to all patients, regardless of pathology or treatment site: 10 minutes in capacitive mode followed by 10 minutes in resistive mode, at low intensity.

The measurements of blood pressure were conducted under standardized conditions, with patients seated and after a minimum of five minutes of rest. Both medical units involved in the study used the same calibrated equipment to ensure consistency. By incorporating these variables into the analysis, it was possible to monitor changes in the patients' resting blood pressure profiles throughout the recovery program, independent of the immediate effects caused by each procedure.

This study monitored changes in both systolic blood pressure (SBP) and diastolic blood pressure (DBP) at several key points within the treatment protocol to assess baseline levels, immediate post-procedure effects, and progression throughout treatment.

Regarding SBP, which reflects the pressure exerted by blood on arterial walls during ventricular contraction and serves as a vital indicator of cardiovascular health, measurements were taken at three distinct points in the protocol, each with specific variables:

SBPz1, SBPz5, SBPz10: SBP values measured prior to starting physiotherapy, on days 1, 5, and 10 of treatment. These provide insights into the patient's baseline blood pressure status.

SBP_T_z1, SBP_T_z5, SBP_T_z10: SBP readings taken immediately after TECAR therapy at the same time points, allowing evaluation of the immediate impact of TECAR on SBP.

SBP_L_z1, SBP_L_z5, SBP_L_z10: SBP values recorded immediately after LLLT therapy, facilitating a direct comparison of the effects between the two physiotherapeutic modalities.

Overall, these measurements trace the patient's blood pressure profile throughout the intervention, establishing benchmarks for analyzing blood pressure changes over time.

Statistical analysis

The data were entered and analyzed using IBM SPSS Statistics for Windows, Version 20 (Released 2011; IBM Corp., Armonk, New York, United States). An initial descriptive analysis was conducted to summarize the study population and the variables included. To evaluate the changes recorded at different time points and examine the cumulative effects of the therapy, repeated-measures analysis of variance (ANOVA) was used. The normality of the data distribution was tested using the Kolmogorov-Smirnov test, which guided the choice of parametric or non-parametric statistical methods. This test determined whether the data followed a normal distribution, a necessary condition for applying parametric tests such as ANOVA. For comparing the effects between normotensive and hypertensive patients, we employed a linear mixed model with random intercepts at the participant level and an unstructured covariance matrix for the repeated measurements across days. When the assumption of normality was violated, non-parametric methods were applied to ensure accurate interpretation of the results. To identify potential predictive factors, multivariate analysis models were constructed. Sphericity was tested using Mauchly’s test; if this assumption was violated (p < 0.05), Greenhouse-Geisser or Huynh-Feldt corrections were applied. Pearson’s correlation coefficient was used to analyze the relationships between continuous variables, provided the data met normality assumptions. All analyses were considered statistically significant at a p-value of < 0.05.

## Results

In total, the study included 268 patients hospitalized in two medical centers: ReumaDiagnostic Clinic (n = 227; 84.7%) and “St. Ioan” Emergency Hospital for Children (n = 41; 15.3%). The age of the participants ranged from 19 to 86 years, with a mean value of 60.3 ± 11.8 years, indicating a cohort composed predominantly of middle-aged and elderly adults. The analysis of patient distribution according to the treated musculoskeletal condition shows that knee osteoarthritis accounted for the largest proportion, representing 28.7% of all investigated cases. It was followed by lumbar disc herniation (17.9%) and cervical disc herniation (12.7%). Other diagnoses recorded with relevant frequency included cervical spondylosis (6.7%), lumbar spondylosis (5.6%), and hip osteoarthritis (4.5%). The overall distribution confirms the predominance of degenerative disorders affecting major joints and the spinal column, reflecting the typical profile of patients requiring rehabilitation therapy. Among the patients included in the study, 66.4% were diagnosed with arterial HTN, while 33.6% did not present this comorbidity. Among the hypertensive patients, 27% had grade III (severe) HTN, 26% had grade II (moderate) HTN, and 13% had grade I (mild) HTN. The high proportion of hypertensive cases highlights the frequent occurrence of this condition among individuals with musculoskeletal disorders, representing a significant risk factor that may influence the physiological response to electrotherapy. Additionally, the 34% of normotensive patients allow for meaningful comparisons between groups with and without HTN regarding blood pressure changes, serving as a potential control group.

In this study, variations in SBP and DBP were monitored at different stages of the therapeutic protocol, aiming to highlight baseline values, immediate post-procedure changes, and the evolution of these parameters throughout the entire treatment period.

Analysis of the data in Table 1 indicates a decreasing trend in SBP following the application of physiotherapeutic procedures. On day 1, the mean SBP declined from 130.37 mmHg before treatment to 127.36 mmHg after TECAR and slightly increased to 129.65 mmHg after LLLT. On day 5, the values remained below the baseline, with 127.84 mmHg pre-treatment, 127.16 mmHg after TECAR, and 128.02 mmHg after LLLT. By day 10, a continued downward trend is evident, with mean SBP values of 127.89 mmHg before treatment, 126.21 mmHg after TECAR, and 126.96 mmHg after LLLT.

**Table 1:** Evolution of systolic blood pressure (mmHg) depending on the day and the therapy TECAR: capacitive and resistive energy transfer therapy; LLLT: low-level laser therapy

Evaluation Day	Before	After TECAR	After LLLT
Day 1	130.37	127.36	129.65
Day 5	127.84	127.16	128.02
Day 10	127.89	126.21	126.96

The diastolic values presented in Table 2 show only slight fluctuations across the three evaluation points. A minor decrease in DBP was observed following TECAR therapy, most evident on day 1 (78.72 mmHg before vs. 78.03 mmHg after TECAR). In contrast, LLLT therapy was linked to a slight increase in diastolic readings, particularly noticeable on day 5 (78.07 mmHg before vs. 81.47 mmHg after LLLT).

**Table 2:** Evolution of diastolic blood pressure (mmHg) depending on the time of evaluation and the type of therapy applied TECAR: capacitive and resistive energy transfer therapy; LLLT: low-level laser therapy

Evaluation Day	Before	According to TECAR	After LLLT
Day 1	78.72	78.03	79.42
Day 5	78.07	78.39	81.47
Day 10	77.44	77.94	78.76

The Kolmogorov-Smirnov test for SBP values was applied at three evaluation time points - day 1 (SBPz1), day 5 (SBPz5), and day 10 (SBPz10), as shown in Table 3. The Asymp. sig. (2-tailed) values were 0.574, 0.109, and 0.204, respectively - all above the 0.05 threshold. This indicates that, at each assessment point, the distribution of blood pressure values approximates a normal distribution, and the null hypothesis of normality is not rejected.

**Table 3:** Kolmogorov-Smirnov test for checking the normality of systolic blood pressure (BP) distribution at baseline assessments ^a^ Test distribution is normal; ^b^ Calculated from data.

One-Sample Kolmogorov-Smirnov Test		Systolic BP Day 1	Systolic BP Day 5	Systolic BP Day 10
N		268	268	268
Normal Parameters^a,b^	Mean	130.37	127.84	12.89
	Hours of deviation	12.267	13.292	11.723
Most Extreme Differences	Absolute	0.048	0.074	0.065
	Positive	0.043	0.073	0.051
	Negative	-0.048	-0.074	-0.065
Kolmogorov-Smirnov Z	-	0.782	1.206	1.068
Asymp. Sig. (2-tailed)	-	0.574	0.109	0.204

The average SBP was 130.37 mmHg on day 1, decreased slightly to 127.84 mmHg on day 5, and remained similar at 127.89 mmHg on day 10. The standard deviations ranged from 11.723 mmHg to 13.292 mmHg, indicating moderate variability among patients. The Most Extreme Differences were minimal, with absolute values between 0.048 and 0.074, further confirming that the data closely follow a Gaussian distribution without significant deviations.

The results of the Kolmogorov-Smirnov test, as shown in Table 4, examine the variables corresponding to SBP measured immediately after TECAR therapy on day 1 (SBP_T_z1), day 5 (SBP_T_z5), and day 10 (SBP_T_z10). The Asymp. sig. (2-tailed) values reveal that the null hypothesis of normality is rejected on day 1 (p = 0.010) and day 5 (p < 0.001), indicating notable deviations from a Gaussian distribution. Conversely, on day 10 (p = 0.122), the data are close to a normal distribution, with no significant differences detected.

**Table 4:** Kolmogorov-Smirnov test for checking the normality of systolic blood pressure (BP) distribution after application of TECAR therapy ^a^ Test distribution is normal; ^b^ Calculated from data; TECAR: capacitive and resistive energy transfer therapy

One-Sample Kolmogorov-Smirnov Test		Systolic BP After TECAR Day 1	Systolic BP After TECAR Day 5	Systolic BP After TECAR Day 10
N		268	268	268
Normal Parameters^a,b^	Mean	127.36	127.16	126.21
	Hours of deviation	14.899	13.651	12.413
Most Extreme Differences	Absolute	0.1	0.133	0.072
	Positive	0.1	0.133	0.072
	Negative	-0.066	-0.062	-0.06
Kolmogorov-Smirnov Z	-	1.633	2.171	1.183
Asymp. Sig. (2-tailed)	-	0.010	0.000	0.122

The mean SBP following TECAR therapy was 127.36 mmHg on day 1, 127.16 mmHg on day 5, and decreased to 126.21 mmHg on day 10. The standard deviations ranged from 12.413 mmHg to 14.999 mmHg, reflecting moderate variability among the measurements. The largest standardized difference (Most Extreme Difference) was observed on day 5 (0.133), confirming a more noticeable deviation from normality at this time point.

The findings shown in Table 5 analyse the distribution of SBP measured immediately after LLLT therapy on day 1 (SBP_L_z1), day 5 (SBP_L_z5), and day 10 (SBP_L_z10). The Asymp. sig. (2-tailed) values were 0.591 for day 1 and 0.189 for day 5, both exceeding the 0.05 threshold, which indicates that the data distribution remains consistent with normality. However, on day 10, the p-value of 0.044 falls below this threshold, suggesting a rejection of the normality assumption and indicating significant deviations from a Gaussian distribution.

**Table 5:** Kolmogorov-Smirnov test for checking the normality of systolic blood pressure (BP) distribution after the application of LLLT therapy ^a^ Test distribution is normal; ^b^ Calculated from data; LLLT: low-level laser therapy

One-Sample Kolmogorov-Smirnov Test		Systolic BP After LLLT Day 1	Systolic BP After LLLT Day 5	Systolic BP After LLLT Day 10
N		268	268	268
Normal Parameters^a,b^	Mean	129.65	128.02	126.96
	Hours of deviation	15.073	13.712	12.652
Most Extreme Differences	Absolute	0.047	0.066	0.084
	Positive	0.047	0.064	0.052
	Negative	-0.03	-0.066	-0.084
Kolmogorov-Smirnov Z	-	0.772	1.086	1.383
Asymp. Sig. (2-tailed)	-	0.591	0.189	0.044

The average SBPs were relatively similar across the time points: 129.65 mmHg on day 1, 128.02 mmHg on day 5, and 126.96 mmHg on day 10. The standard deviations ranged from 12.652 mmHg to 15.073 mmHg, reflecting moderate variability among participants. The Most Extreme Differences values were minimal, with a maximum of 0.084 on day 10, reinforcing the observation of a slight deviation from normality at this time point

Comparative interpretation

The comparative analysis indicates that TECAR therapy produced a faster and more significant hypotensive effect, particularly on the first day of treatment. However, this response exhibited greater variability among patients. Conversely, LLLT therapy results in smaller, but consistent reductions in SBP, with a more uniform distribution of values during the initial two measurement points.

The comparative analysis of DBP values shows a stable evolution of DBP before therapy sessions, with a slight decrease throughout the treatment, suggesting the preservation of physiological balance. Following TECAR therapy, immediate variations were minimal, with an initially normal distribution and a slight increase in variability toward the end, indicating individual differences in response. In the case of LLLT, a slight rise in diastolic values was observed, most evident on day 5, when significant deviation from normality and increased variability suggested the presence of atypical responses.

Systolic blood pressure (SBP)

A repeated measures ANOVA was used, as it is appropriate for studies in which the same participants are evaluated at different stages of the intervention. This analysis allows comparison of mean values over time and identification of significant differences between assessment points.

Table 6 illustrates the organization of the dependent variables based on the two intra-subject factors incorporated in the repeated measures ANOVA model. The "Time" factor represents the immediate changes induced by the electrotherapy procedures (values before treatment, after TECAR application, and after LLLT application), while the "Day" factor reflects the variations across the three assessment points (day 1, day 5, and day 10).

**Table 6:** Structure of intra-subject factors for the analysis of systolic blood pressure variations

Time	Day	Dependent Variable
1	1	SBPz1
	2	SBP_T_z1
	3	SBP_L_z1
2	1	SBPz5
	2	SBP_T_z5
	3	SBP_L_z5
3	1	SBPz10
	2	SBP_T_z10
	3	SBP_L_z10

The interpretation of the results in Table 7 reveals statistically significant effects for both the Time factor (Pillai's Trace = 0.053; F(2,266) = 7.479; p = 0.001) and the Day factor (Pillai's Trace = 0.066; F(2,266) = 9.436; p < 0.001). These findings confirm that SBP values vary significantly depending on the timing of the electrotherapy procedures (before, after TECAR, and after LLLT) and also fluctuate across the three assessment points (day 1, day 5, and day 10).

**Table 7:** Multivariate tests for the effects of intra-subject factors (Time, Day and Time×Day interaction) on systolic blood pressure ^b^ Statistical significance

Effect		Value	F	Hypothesis df	Error df	Itself.
Time	Pillai's Trace	0.053	7.479^b^	2	266	0.001
	Wilks' Lambda	0.947	7.479^b^	2	266	0.001
	Hotelling's Trace	0.056	7.479^b^	2	266	0.001
	Roy's Largest Root	0.056	7.479^b^	2	266	0.001
Day	Pillai's Trace	0.066	9.436^b^	2	266	0.000
	Wilks' Lambda	0.934	9.436^b^	2	266	0.000
	Hotelling's Trace	0.071	9.436^b^	2	266	0.000
	Roy's Largest Root	0.071	9.436^b^	2	266	0.000
Time * Day	Pillai's Trace	0.05	3.444^b^	4	264	0.009
	Wilks' Lambda	0.95	3.444^b^	4	264	0.009
	Hotelling's Trace	0.052	3.444^b^	4	264	0.009
	Roy's Largest Root	0.052	3.444^b^	4	264	0.009

Additionally, the interaction between Time and Day is statistically significant (Pillai's Trace = 0.050; F(4.264) = 3.444; p = 0.009), indicating that the impact of electrotherapy on SBP varies depending on the day the measurements were taken.

The results in Table 8 demonstrate that the sphericity assumption is violated for the Time factor (W = 0.967; χ^2^(2) = 8.848; p = 0.012), the Day factor (W = 0.942; χ^2^(2) = 15.991; p < 0.001), and their interaction (W = 0.761; χ^2^(9) = 72.417; p < 0.001). This suggests that the assumption of equal covariance matrices is not satisfied, which may influence the validity of standard univariate tests.

**Table 8:** Mauchly test of sphericity for intra-subject factors (Time, Day, and Time×Day interaction) ^b^ May be used to adjust the degrees of freedom for the averaged tests of significance.

Within Subjects Effect	Mauchly's W	Approx. Chi-Square	df	Itself.	Epsilon^b^		
					Greenhouse-Geisser	Huynh-Feldt	Lower-bound
Time	0.967	8.848	2	0.012	0.968	0.975	0.5
Day	0.942	15.991	2	0.000	0.945	0.951	0.5
Time * Day	0.761	72.417	9	0.000	0.89	0.904	0.25

In order to correct the degrees of freedom and obtain valid results, the Greenhouse-Geisser and Huynh-Feldt adjustments were used, which provide robust estimates in the presence of sphericity violations. Consequently, the final interpretation of intra-subject effects will be based on these corrections, according to Table 9.

**Table 9:** Intra-subject effects tests for systolic blood pressure values

Source		Type III Sum of Squares	df	Mean Square	F	Itself.
Time	Sphericity Assumed	1873.483	2	936.7	8.7	0.000
	Greenhouse-Geisser	1873.483	2	967.4	8.7	0.000
	Huynh-Feldt	1873.483	2	960.5	8.7	0.000
	Lower-bound	1873.483	1	1873.5	8.7	0.003
Error(Time)	Sphericity Assumed	57277.41	534	107.3	-	-
	Greenhouse-Geisser	57277.41	517	110.8	-	-
	Huynh-Feldt	57277.41	521	110.0	-	-
	Lower-bound	57277.41	267	214.5	-	-
Day	Sphericity Assumed	1374.095	2	687.0	11.5	0.000
	Greenhouse-Geisser	1374.095	2	727.1	11.5	0.000
	Huynh-Feldt	1374.095	2	722.1	11.5	0.000
	Lower-bound	1374.095	1	1374.1	11.5	0.001
Error(day)	Sphericity Assumed	31855.46	534	59.7	-	-
	Greenhouse-Geisser	31855.46	505	63.1	-	-
	Huynh-Feldt	31855.46	508	62.7	-	-
	Lower-bound	31855.46	267	119.3	-	-
Time * Day	Sphericity Assumed	441.828	4	110.5	3.5	0.008
	Greenhouse-Geisser	441.828	4	124.1	3.5	0.011
	Huynh-Feldt	441.828	4	122.2	3.5	0.010
	Lower-bound	441.828	1	441.8	3.5	0.063
Error(Time*Day)	Sphericity Assumed	33952.62	1068	31.8	-	-
	Greenhouse-Geisser	33952.62	951	35.7	-	-
	Huynh-Feldt	33952.62	965	35.2	-	-
	Lower-bound	33952.62	267	127.2	-	-

The analysis of the results in Table 9 indicates that the Time factor has a significant influence on SBP values (F(2.534) = 8.7, p < 0.001), confirming that there are notable differences between the measurements taken before therapy, after TECAR application, and after LLLT.

Furthermore, the Day factor also has a significant impact (F(2.534) = 11.5, p < 0.001), demonstrating that SBP values change systematically across the three assessment points (day 1, day 5, and day 10).

The results presented in Table 10 show that the model intercept is highly statistically significant, F(1.267) = 34594.88, p < 0.001. This indicates that the mean SBP value differs substantially from zero, and the variations observed reflect a genuine effect rather than random fluctuations.

**Table 10:** Between-subjects effects test for mean systolic blood pressure values

Source	Type III Sum of Squares	df	Mean Square	F	Sig.
Intercept	39481096.72	1	39481097	34594.88	0.000
Error	304711.394	267	1141.241	-	-

The residual error is relatively small compared with the magnitude of the intercept effect, indicating low variability in measurements across participants. This finding strengthens the stability of the model and supports the reliability of the conclusions regarding the influence of within-subject factors on blood pressure values.

Diastolic blood pressure (DBP)

The data summarized in Table 11 show the organization of the dependent variables used in the ANOVA analysis for repeated measures. The "Time" factor includes the three evaluation stages (before therapy, after TECAR application, and after LLLT application), and the "Day" factor captures their dynamics in the three measurement moments (day 1, day 5, and day 10).

**Table 11:** Structure of intra-subject factors for the analysis of variations in diastolic blood pressure

Time	Day	Dependent Variable
1	1	DBPz1
	2	DBP_T_z1
	3	DBP_L_z1
2	1	DBPz5
	2	DBP_T_z5
	3	DBP_L_z5
3	1	DBPz10
	2	DBP_T_z10
	3	DBP_L_z10

This structure allows for the analysis of variations in DBP in relation to the type of intervention and its progression over time, as well as the evaluation of the interaction between the two factors.

According to the results presented in Table 12, neither the Time factor (Pillai's Trace = 0.017; F(2.266) = 2.256; p = 0.107), the Day factor (Pillai's Trace = 0.011; F(2.266) = 1.478; p = 0.230), nor their interaction (Pillai's Trace = 0.016; F(4.264) = 1.096; p = 0.359) reached the conventional threshold for statistical significance.

**Table 12:** Multivariate tests for the effects of intra-subject factors (Time, Day and Time×Day interaction) on diastolic blood pressure ^b^ Statistically accurate

Effect						Value			F		Hypothesis df	Error df	Itself.
Time	Pillai's Trace					0.017			2.256^b^		2	266	0.107
	Wilks' Lambda					0.983			2.256^b^		2	266	0.107
	Hotelling's Trace					0.017			2.256^b^		2	266	0.107
	Roy's Largest Root					0.017			2.256^b^		2	266	0.107
Day	Pillai's Trace					0.011			1.478^b^		2	266	0.230
	Wilks' Lambda					0.989			1.478^b^		2	266	0.230
	Hotelling's Trace					0.011			1.478^b^		2	266	0.230
	Roy's Largest Root					0.011			1.478^b^		2	266	0.230
Time * Day	Pillai's Trace					0.016			1.096^b^		4	264	0.359
	Wilks' Lambda					0.984			1.096^b^		4	264	0.359
	Hotelling's Trace					0.017			1.096^b^		4	264	0.359
	Roy's Largest Root					0.017			1.096^b^		4	264	0.359

The results suggest that DBP remains relatively stable, showing no significant variations related to the application of TECAR and LLLT therapies, the timing of evaluations, or the interaction of these factors, with the hypothesis of significant changes not being statistically supported.

According to the results presented in Table 13, the sphericity hypothesis is rejected for all the factors analyzed: Time (W = 0.271; χ^2^(2) = 347.65; p < 0.001), Day (W = 0.329; χ^2^(2) = 295.344; p < 0.001) and Time × Day interaction (W = 0.003; χ^2^(9) = 1522.792; p < 0.001).

**Table 13:** Mauchly test of sphericity for intra-subject factors (Time, Day, and Time×Day interaction) on diastolic blood pressure ^b^ May be used to adjust the degrees of freedom for the averaged tests of significance.

Within Subjects Effect	Mauchly's W	Approx. Chi-Square	df	Itself.	Epsilon^b^		
					Greenhouse-Geisser	Huynh-Feldt	Lower-bound
Time	0.271	347.65	2	0.000	0.578	0.579	0.5
Day	0.329	295.344	2	0.000	0.599	0.6	0.5
Time * Day	0.003	1522.792	9	0.000	0.291	0.291	0.25

This suggests that the variances and covariances are not equal across conditions, which violates the assumption of sphericity. To address this issue and ensure valid results, the Greenhouse-Geisser and Huynh-Feldt corrections were applied, based on the epsilon values provided in the table.

The results in Table 14 demonstrate that the Time factor does not have a statistically significant effect on DBP values (F(2.534) = 1.002, p = 0.368). This indicates that the measurements taken before therapy, after TECAR application, and after LLLT application do not differ significantly from each other.

**Table 14:** Intra-subject effects tests for diastolic blood pressure values

Source		Type III Sum of Squares	df	Mean Square	F	Itself.
Time	Sphericity Assumed	641	2	320.3	1.002	0.368
	Greenhouse-Geisser	641	1	553.8	1.002	0.329
	Huynh-Feldt	641	1	552.9	1.002	0.329
	Lower-bound	641	1	640.5	1.002	0.318
Error(Time)	Sphericity Assumed	170717	534	319.7	-	-
	Greenhouse-Geisser	170717	309	552.9	-	-
	Huynh-Feldt	170717	309	552.0	-	-
	Lower-bound	170717	267	639.4	-	-
Day	Sphericity Assumed	1706	2	852.9	2.649	0.072
	Greenhouse-Geisser	1706	1	1424.8	2.649	0.098
	Huynh-Feldt	1706	1	1422.0	2.649	0.098
	Lower-bound	1706	1	1705.8	2.649	0.105
Error(Day)	Sphericity Assumed	171950	534	322.0	-	-
	Greenhouse-Geisser	171950	320	537.9	-	-
	Huynh-Feldt	171950	320	536.9	-	-
	Lower-bound	171950	267	644.0	-	-
Time * Day	Sphericity Assumed	680	4	170.1	0.577	0.679
	Greenhouse-Geisser	680	1	584.9	0.577	0.473
	Huynh-Feldt	680	1	584.0	0.577	0.473
	Lower-bound	680	1	680.4	0.577	0.448
Error(Time*Day)	Sphericity Assumed	314838	1068	294.8	-	-
	Greenhouse-Geisser	314838	311	1013.8	-	-
	Huynh-Feldt	314838	311	1012.1	-	-
	Lower-bound	314838	267	1179.2	-	-

For the Day factor, a non-significant trend was observed, F(2.534) = 2.649, p = 0.072, indicating that changes in DBP between days 1, 5, and 10 do not reach the conventional threshold for statistical significance.

Similarly, the Time × Day interaction was not significant, F(4.1068) = 0.577, p = 0.679, suggesting that the effect of physiotherapeutic procedures on DBP does not vary substantially depending on the timing of the evaluation. Overall, the data suggest that, unlike SBP values, diastolic readings did not show significant changes following TECAR and LLLT treatments.

Blood pressure response characteristics in hypertensive versus normotensive patients in the context of electrotherapy

The blood pressure response to electrotherapeutic procedures such as TECAR and LLLT may be influenced by the patient’s baseline blood pressure status. Comparing individuals with HTN to those with normal blood pressure provides valuable insight into the safety and applicability of these therapies in cardiovascularly vulnerable populations.

The following analysis aims to compare the magnitude of SBP reduction after TECAR and LLLT therapies between patients with known HTN and normotensive individuals, in order to assess whether pre-existing cardiovascular status influences the physiological response to treatment.

The dependent variable will be the combined daily effect, calculated across the three assessment points. Fixed effects will include the HTN group, the evaluation day, and the interaction between group and day. The main analysis will focus on the combined effect, used as the dependent variable to capture the overall impact of the electrotherapeutic session on blood pressure.

Table 15 illustrates variations in the immediate impact of TECAR therapy on SBP, showing distinct response patterns between hypertensive and normotensive patients at the three evaluation time points.

**Table 15:** Descriptive statistics for the immediate effect of TECAR therapy on systolic blood pressure (mmHg), according to hypertension status, on days 1, 5, and 10 TECAR: capacitive and resistive energy transfer therapy

Descriptive Statistics	Hypertension	Mean	Std. Deviation	N
the immediate effect of TECAR_ day1	No	4.72	10.231	90
	Yes	2.15	11.081	178
	Total	3.01	10.852	268
the immediate effect of TECAR_day5	No	-1.20	9.172	90
	Yes	1.62	10.486	178
	Total	0.68	10.135	268
the immediate effect of TECAR_day10	No	1.23	6.655	90
	Yes	1.90	8.435	178
	Total	1.68	7.876	268

On day 1, normotensive patients experienced a more pronounced decrease in SBP (M = 4.72 mmHg, SD = 10.23) than hypertensive patients (M = 2.15 mmHg, SD = 11.08), indicating a quicker initial response to TECAR therapy. By day 5, this pattern reversed, with hypertensive patients showing a modest reduction (M = 1.62 mmHg, SD = 10.48) and normotensive patients a slight increase (M = -1.20 mmHg, SD = 9.17), suggesting differing cardiovascular adaptation mechanisms. By day 10, both groups exhibited minimal and comparable changes (hypertensive: M = 1.90 mmHg, SD = 8.43; normotensive: M = 1.23 mmHg, SD = 6.65), indicating short-term stabilization and convergence of the blood pressure response to TECAR therapy.

The multivariate results presented in Table 16 reveal a significant main effect of the Day factor on the immediate change in SBP after TECAR therapy. The consistent test statistics (Pillai’s Trace = 0.058; Wilks’ Lambda = 0.942; Hotelling’s Trace = 0.062; Roy’s Largest Root = 0.062) and the F value (F(2,265) = 8.159, p < 0.001) indicate that the blood pressure response varied across the evaluation days (Day 1, Day 5, and Day 10). The observed power (0.958) suggests a high probability of detecting a true effect.

**Table 16:** Multivariate tests for the effect of the “Day” factor and the “Day × Hypertension” interaction on the immediate TECAR effect ^b^ Exact statistic; ^c^ Computed using alpha = 0.05 TECAR: capacitive and resistive energy transfer therapy; HTN: hypertension

Effect		Value	F	Hypothesis df	Error df	Sig.	Noncent. Parameter	Observed Power^c^
Day	Pillai's Trace	0.058	8.159^b^	2	265	0.000	16.318	0.958
	Wilks' Lambda	0.942	8.159^b^	2	265	0.000	16.318	0.958
	Hotelling's Trace	0.062	8.159^b^	2	265	0.000	16.318	0.958
	Roy's Largest Root	0.062	8.159^b^	2	265	0.000	16.318	0.958
Day * HTN	Pillai's Trace	0.041	5.714^b^	2	265	0.004	11.427	0.862
	Wilks' Lambda	0.959	5.714^b^	2	265	0.004	11.427	0.862
	Hotelling's Trace	0.043	5.714^b^	2	265	0.004	11.427	0.862
	Roy's Largest Root	0.043	5.714^b^	2	265	0.004	11.427	0.862

The Day × HTN interaction was statistically significant (F(2,265) = 5.714, p = 0.004), indicating distinct TECAR response patterns between hypertensive and normotensive patients. The Pillai’s Trace (0.041) and observed power (0.862) confirm a strong link, suggesting that baseline cardiovascular status shapes the physiological response to therapy.

The results presented in Table 17 show that the incremental effect of LLLT on SBP is reflected by negative values, indicating an additional average reduction in blood pressure following LLLT, beyond the decrease already achieved with TECAR therapy.

**Table 17:** Descriptive statistics for the incremental effect of LLLT therapy on systolic blood pressure (mmHg), according to hypertension status LLLT: low-level laser therapy

Descriptive Statistics	Hypertension	Mean	Std. Deviation	N
Incremental effect of LLLT day 1	No	-1.99	6.89	90
	Yes	-2.45	10.61	178
	Total	-2.29	9.51	268
Incremental effect of LLLT day 5	No	-1.10	9.04	90
	Yes	-0.74	8.41	178
	Total	-0.86	8.62	268
Incremental effect of LLLT​​​​​​​ day 10	No	-0.40	5.13	90
	Yes	-0.92	8.92	178
	Total	-0.75	7.85	268

On day 1, hypertensive patients showed a slightly greater reduction (M = -2.45 mmHg, SD = 10.61) than normotensive ones (M = -1.99 mmHg, SD = 6.89). By day 5, the values were comparable, with normotensive patients showing a marginally larger decrease (M = -1.10 mmHg) than hypertensive patients (M = -0.74 mmHg). On day 10, both groups displayed minimal incremental effects (hypertensive: M = -0.92 mmHg; normotensive: M = -0.40 mmHg), indicating a gradual attenuation of LLLT’s contribution to SBP reduction as treatment progressed. Overall, LLLT produced a modest additional decrease in blood pressure, more pronounced during the initial sessions, with minimal differences between groups, suggesting a generally uniform incremental effect regardless of HTN status.

The results in Table 18 show a significant main effect of the Day factor on the incremental change in SBP after LLLT (Pillai’s Trace = 0.023; Wilks’ Lambda = 0.977; F(2,265) = 3.111; p = 0.046). This indicates that the additional effect of LLLT varies modestly but significantly across evaluation days (Day 1, Day 5, and Day 10). However, the observed power (0.595) falls below the recommended threshold of 0.80, suggesting only moderate sensitivity for detecting the true effect.

**Table 18:** Multivariate tests for the effect of the “Day” factor and the “Day × HTN” interaction on the incremental effect of LLLT ^b^ Exact statistic; ^c^ Computed using alpha = 0.05 LLLT: low-level laser therapy

Effect		Value	F	Hypothesis df	Error df	Sig.	Noncent. Parameter	Observed Power^c^
Day	Pillai's Trace	0.023	3.111^b^	2	265	0.046	6.222	0.595
	Wilks' Lambda	0.977	3.111^b^	2	265	0.046	6.222	0.595
	Hotelling's Trace	0.023	3.111^b^	2	265	0.046	6.222	0.595
	Roy's Largest Root	0.023	3.111^b^	2	265	0.046	6.222	0.595
Day * HTN	Pillai's Trace	0.002	0.321^b^	2	265	0.726	0.642	0.101
	Wilks' Lambda	0.998	0.321^b^	2	265	0.726	0.642	0.101
	Hotelling's Trace	0.002	0.321^b^	2	265	0.726	0.642	0.101
	Roy's Largest Root	0.002	0.321^b^	2	265	0.726	0.642	0.101

The Day × HTN interaction was not statistically significant (F(2,265) = 0.321, p = 0.726, power = 0.101), suggesting that changes in the incremental effect of LLLT followed a comparable pattern in both hypertensive and normotensive groups, without meaningful differences between them.

The combined effect of TECAR and LLLT therapy on SBP showed a mild, fluctuating, and transient response, with no marked differences between hypertensive and normotensive patients. Descriptive statistics indicate that in the initial sessions, responses varied by HTN status - normotensive patients showed a slight increase, while hypertensive patients exhibited a modest decrease. By the fifth session, this pattern reversed, and by the 10th, mean values converged, suggesting progressive adaptation and diminishing group differences.

## Discussion

The findings of this study indicate modest and transient fluctuations in SBP during the applied therapeutic protocol, with a slightly more pronounced reduction observed following TECAR compared with LLLT. Statistical analyses showed a significant Time effect, highlighting differences between measurements recorded before treatment, immediately after TECAR, and immediately after LLLT. Although these differences reached statistical significance, their magnitude was small and within the range of normal biological variability. The Day factor also demonstrated systematic variation across the three evaluation points (days 1, 5, and 10), while the significant Time × Day interaction suggested that the observed changes depend on the phase of the treatment protocol in which the intervention is applied. Nevertheless, these fluctuations - despite being statistically detectable - lack clinical relevance.

The slight, short-term reduction in SBP was more pronounced after TECAR, a finding potentially attributable to its local physiological effects, including enhanced microcirculation and reduced peripheral vascular resistance via localized vasodilation. The absence of significant changes in DBP further suggests that these responses remain predominantly local, without evidence of systemic hemodynamic modulation. This pattern supports a cautious interpretation: TECAR may elicit a transient systolic decrease more evident than that observed with LLLT, yet the overall effect remains small, non-sustained, and clinically negligible.

When comparing hypertensive and normotensive patients, SBP changes followed a similar overall trajectory, with small, fluctuating differences in the early sessions and convergence of values by the end of the treatment period. Although normotensive patients can serve as a limited exploratory comparison group, this approach does not substitute for a true control arm and does not allow for causal attribution of the observed changes to the applied interventions.

Monitoring blood pressure throughout the protocol served two essential purposes: ensuring cardiovascular safety by identifying possible transient responses, and enabling analytical comparison - particularly through contrasting immediate post-LLLT values with corresponding post-TECAR measurements at the same evaluation points. The overall stability of blood pressure values, especially diastolic measurements, reinforces the conclusion that these procedures are well tolerated from a cardiovascular standpoint.

These results align with the existing literature, which predominantly documents local - rather than systemic - physiological effects of TECAR and LLLT. Tashiro et al. (2017) demonstrated increases in oxyhemoglobin and total hemoglobin following TECAR, associated with vasodilation and improved microcirculation [9]. Clijsen et al. (2020) similarly reported enhanced intramuscular blood flow without any changes in blood pressure or heart rate, supporting the notion of a localized effect without systemic cardiovascular impact [10]. Kumaran and Watson observed sustained increases in skin temperature and cutaneous blood flow after radiofrequency therapy, while heart rate and blood pressure remained unchanged [11].

Evidence regarding systemic cardiovascular effects of LLLT remains limited. Most studies have been performed in vitro or on animal models, with human research still in its early stages. Sayed et al. (2023), investigating 27 patients with chronic systolic heart failure (New York Heart Association (NYHA) III/IV), reported improvements in ejection fraction and NYHA class after 10 sessions of LLLT applied to acupuncture points, but without meaningful alterations in diastolic function [12]. Zhang et al. documented reductions in both SBP and DBP in hypertensive patients following a combined acupuncture-LLLT intervention; however, the multimodal nature of the treatment prevents attribution of these effects solely to LLLT [13]. Animal studies further suggest potential cardiovascular effects, yet their translational relevance remains uncertain [8].

Overall, the present findings do not support a significant systemic impact of TECAR or LLLT on arterial blood pressure but reinforce the cardiovascular safety of these modalities. The systolic changes observed - although statistically identifiable - are small and transient.

Limitations

This study has several methodological and experimental limitations that should be considered when interpreting the findings. The absence of randomization, blinding, and a control or sham group prevents the establishment of causal relationships between the application of TECAR and LLLT therapies and the observed blood pressure changes, and factors such as regression to the mean or measurement-related effects cannot be fully excluded.

Another important limitation is that blood pressure was assessed at discrete time points rather than through continuous ambulatory monitoring (e.g., Holter blood pressure recording). This approach restricts the ability to capture circadian variations, transient fluctuations, and potential immediate or rebound effects following treatment.

The study also did not include the evaluation of biomarkers related to oxidative stress, inflammation, or endothelial function (such as high-sensitivity C-reactive protein (hs-CRP), interleukin-6 (IL-6), and tumor necrosis factor-alpha (TNF-α)), which could have provided additional insight into the underlying physiological mechanisms. Although the sample size is moderate, both the number of participants and the relatively short follow-up period warrant caution when generalizing the results.

Overall, these limitations do not invalidate the descriptive observations obtained but call for a cautious interpretation and underscore the need for future randomized clinical trials with larger samples, continuous hemodynamic monitoring, and biomolecular assessments to confirm potential causal relationships and strengthen the evidence regarding the safety and efficacy of these therapies.

## Conclusions

The analysis of SBP and DBP values shows that hemodynamic parameters in patients remained relatively stable throughout the physiotherapy protocol. Electrotherapy proved to be cardiovascularly safe, with no significant variations in blood pressure observed. At the same time, the differences identified between TECAR and LLLT therapies suggest that each treatment type may influence the hemodynamic response in distinct ways, highlighting the need for further research through clinical studies on larger patient groups and continuous monitoring of cardiovascular parameters.
